# Neurological Outcome of Spinal Hemangioblastomas: An International Observational Multicenter Study About 35 Surgical Cases

**DOI:** 10.3390/cancers17091428

**Published:** 2025-04-24

**Authors:** Motaz Alsereihi, Donato Creatura, Ginevra F. D’Onofrio, Alberto Vandenbulcke, Mahmoud Messerer, Nicolas Penet, Raul Lozano-Madrigal, Alberto Delaidelli, Federico Pessina, Gabriele Capo, Cédric Y. Barrey

**Affiliations:** 1 Department of Spine and Spinal Cord Surgery, Hôpital Pierre Wertheimer, GHE, Hospices Civils de Lyon, Claude Bernard University of Lyon 1, 59 Boulevard Pinel, 69677 Lyon, France; motaz.alsereihi@chu-lyon.fr (M.A.); donato.creatura@chu-lyon.fr (D.C.); 2 Department of Surgery, Umm Al-Qura University, Mecca 24382, Saudi Arabia; 3 Department of Neurosurgery, IRCSS Humanitas Research Hospital, 20089 Milan, Italy; rlm_88@hotmail.com (R.L.-M.); federico.pessina@hunimed.eu (F.P.); gabriele.capo@humanitas.it (G.C.); 4 Department of Neurosurgery, Policlinico Agostino Gemelli Hospital, 00168 Rome, Italy; ginevra.federica.donofrio@gmail.com; 5 Department of Neurosurgery, Lausanne University Hospital, CH-1011 Lausanne, Switzerland; alberto.vandenbulcke@gmail.com (A.V.); mahmoud.messerer@chuv.ch (M.M.); nicolas.penet@chuv.ch (N.P.); 6 Department of Molecular Oncology, British Columbia Cancer Research Centre, Vancouver, BC V5Z 1L3, Canada; adelaidelli@bccrc.ca; 7 Department of Pathology and Laboratory Medicine, University of British Columbia, Vancouver, BC V6T 1Z7, Canada; 8 Department of Biomedical Sciences, Humanitas University, 20072 Milan, Italy; 9 Laboratory of Biomechanics, ENSAM, Arts et Metiers ParisTech, 153 Boulevard de l’Hôpital, 75013 Paris, France

**Keywords:** hemangioblastoma, spinal cord, clinical outcome, neurological outcome, Von Hippel–Lindau, neurosurgery

## Abstract

Introduction: Hemangioblastomas (HBs) are benign, highly vascular tumors that can be found intracranially or in the spinal region, representing around 2–15% of primary intramedullary tumors. They can occur sporadically or in association with Von Hipple–Lindau (VHL) disease. Despite recent of advancement of nonsurgical treatments, complete surgical resection remains the gold standard of care for the spinal HBs. Materials and Methods: We conducted an international multicenter retrospective analysis of adult patients surgically treated for spinal HBs in four European referral centers between January 2000 and September 2024, with a minimum post-operative follow-up duration of 6 months. Patients’ sex and age at surgical intervention, clinical presentation, and duration symptoms prior to clinical diagnosis were identified. The pre- and post-operative neurological status at 1 and 6 months and at the last visit was assessed using the modified McCormick score (MCS). The extent of surgical resection was divided into gross total resection (GTR) and subtotal resection (STR). Finally, post-operative complications were inspected as well, namely cerebrospinal fluid leaks, infections, hemorrhages and post-operative spinal stability. Results: A total of 35 patients were included in the cohort, with an age median of 52 years (34.5–60) and a slight male predominance (21/35, i.e., 60%). The median follow-up period was 37.5 months (12–75). More than half were located in the cervical region, making it the most common (54.3%). Syrinxes were observed in 23 cases (72%), and HBs were more commonly intramedullary (80%). GTR was achievable in around 88% of cases. Post-operative complications were observed in nine patients (25.7%). Nearly half of patients were discharged into rehabilitations centers (48.5%). Tumor recurrence was seen in 10.3% only. At the last follow-up, an excellent overall post-operative neurological status (positive ∆ McCormick) was observed in most of patients (88%) and was found to be associated with a relatively younger age group. Tumor location and presence of syrinxes did not show any statistical significance regarding clinical outcome. In patients having benefited from intra-operative monitoring, only D-wave changes showed statistical significance regarding post-operative outcome (*p* < 0.05). Conclusions: A large majority of patients operated for a spinal HB demonstrated favorable outcome after surgery, with unchanged or improved neurological status. Advanced age could have an impact on the post-operative neurological outcome. Other factors such as tumor size, location, and the presence of syrinx did not seem to significantly impact the neurological outcome. Finally, the surgery of these vascular lesions with no possibility of debulking or piece-meal removal and requiring “en bloc” resection is technically demanding and should be performed by experienced teams in spine and spinal cord surgery only.

## Simple Summary

Hemangioblastomas (HBs) are benign highly vascular tumors representing around 2–15% of primary intramedullary tumors and can occur sporadically or in association with Von Hipple Lindau (VHL) disease. Despite recent of advancement of nonsurgical treatments, complete surgical resection remains the gold standard of care for the spinal HBs. An international multicenter observational study of adult patients surgically treated for spinal HBs was carried out in four European referral centers between January 2000 and September 2024. A total of 35 patients were included in the cohort with an age median of 52 years and a median follow-up period of 37.5 months. GTR was achievable in around 88% of cases with tumor recurrence seen in 10.3%. A large majority of patients operated for a spinal HB demonstrated favorable outcome after surgery with unchanged or improved neurological status. Advanced age could have a negative impact on the post operative neurological outcome. Other factors such as tumor size, location and the presence of syrinx did not seem to significantly impact the neurological outcome. Surgical treatment of these vascular lesions with no possibility of debulking or piece-meal removal is technically demanding and should be performed by experienced teams in spine and spinal cord surgery.

## 1. Introduction

Hemangioblastomas (HBs) are benign, highly vascular tumors classified as grade 1 according to the World Health Association (WHO) classification [1,2,3,4,5]. They are more commonly found in the intracranial compartment, mainly in the posterior fossa, but can be found in the spinal region as well [6]. Spinal HBs may be intramedullary or, less commonly, extramedullary or may have a combined intra/extramedullary form [2,7]. These lesions are rare and represent only 2–6% of all spinal tumors and around 2–15% of primary intramedullary spinal cord tumors and are much less commonly occurring than astrocytomas and ependymomas [3,8,9]. HBs can occur sporadically (70–80%) or in association with Von Hipple–Lindau (VHL) disease (20–30%), an autosomal dominant disorder characterized by a germline mutation in the VHL gene located in the chromosome 3p25–26 region [3,8,10,11,12,13]. VHL disease is associated with several other neoplasms, such pheochromocytoma and renal cell carcinoma [3]. The incidence of this condition is estimated to be 1 in 36,000 newborns [6,10]. A slight male predominance has been observed at a ratio of 1.6:1 to 5.5:1 [14].

Clinical manifestations resulting from spinal HBs depend on their size, location, and the presence of syringomyelia and spinal cord edema. These manifestations can vary from general pain and discomfort to significant neurological deficits affecting patients’ quality of the life [2,5,8].

Despite the occasional use of angiographic studies, magnetic weighted imaging (MRI) remains the gold standard when it comes to radiological evaluation [6,15,16]. Spinal HBs can vary with regards to their signal intensities on T1- and T2-weighted images (WI). On T1WI, they are typically iso- or hypointense. On the other hand, they are usually either hyper- or isointense on T2WI. With regards to gadolinium enhancement, these often show a nodular enhancing pattern on T1WI+ [6,16,17].

Despite recent advancements in nonsurgical treatments, such as fractionated and stereotaxic radiosurgery, as well as medical treatments such as Belzutifan in the context of VHL, complete surgical resection remains the gold standard of care for the spinal hemangioblastoma, with a good overall outcome [1,6,9,18,19,20,21]. Complete surgical resection is achievable in most cases, as demonstrated in recent studies, with subtotal resection needing a second surgery being relatively uncommon [1,2]. However, the use of adjuvant radiotherapy is also considered in case of recurrence or in difficulty achieving complete surgical excision [4,20,22].

Due to the rarity of these tumors, few relatively large case series have been published. Furthermore, even fewer multicentric international studies analyzing more than 20 cases can be found in the literature [2,23,24], and the results provided in these are mostly heterogenous. In this context, the aim of the present study was to report results from an international multicenter cohort, with a special focus on the management and neurological outcome of patients with histologically confirmed spinal HBs.

## 2. Material and Method

### 2.1. Study Design and Patients’ Population

After obtaining the ethical approval by the scientific and ethics committee (Scientific and Ethical Committee of Hospices Civils de Lyon, IRB 00013204, ethical approval no. 23-5395), we conducted an international retrospective analysis of adult patients followed and treated for pathologically confirmed spinal hemangioblastomas, in BLINDED CENTERS. All adult patients surgically treated in these institutions between January 2000 and September 2024, with a minimum post-operative follow-up duration of 6 months, were included in the study.

### 2.2. Patient Evaluation

Patients’ sex and age at surgical intervention, clinical presentation, and duration symptoms prior to clinical diagnosis were identified. A thorough clinical evaluation was carried out immediately before and after surgical intervention. The Frankel score was used to assess patients’ pre-operative neurological status, while post-operative neurological status was assessed using the modified McCormick score (MCS) for the sake of comparison (Table 1). A minimum follow-up duration of six months was ensured for all patients. We also addressed the neurological status at 1 and 6 months and at the last visit, when available, to properly investigate the post-operative surgical outcome and disease progression. Patients with follow-up periods less than six months were excluded from the study.

### 2.3. Radiological Evaluation

Radiologically, we inspected pre- and post-operative MRIs, with focus on T1WI with gadolinium contrast, T2WI, and FLAIR, analyzing tumor size and location, the presence of syringomyelia, as well as the use of pre-operative digital subtraction angiography (DSA). Tumor size was assessed based on the largest height, width, and length in mm. Lesions were classified into purely intramedullary, purely extramedullary, or combined intra/extramedullary in relation to the spinal cord. They were also classified into ventral/ventrolateral, dorsal/dorsolateral, and central based on their transversal location. The cranio-caudal location was noted and specified into cervical, cervico-dorsal, dorsal, dorso-lumbar, and lumbar. Post-operative MRI was also used to assess degree of resection and recurrence. The number of spinal levels affected was also evaluated (1–2 versus ≥ 3). T1WI with contrast was used to detect tumor presence, and T2WI and FLAIR were used for the evaluation of cystic changes.

### 2.4. Extent of Surgical Resection

The surgical data were divided based on the extent of resection into gross total resection (GTR) and subtotal resection (STR). The surgery was conducted via standard posterior midline myelotomy for purely intra-medullary lesions and via direct approach for exophytic tumors. The principle was to devascularize the lesion by dissecting the feeding arteries at its surface avoiding penetrating inside the tumor. The draining vein was sacrificed at the end of the surgical procedure, as is usual for vascular lesions. Complications related to the surgery were inspected as well, namely neurological deterioration, cerebrospinal fluid leaks, infections, hemorrhages, and post-operative spinal instability.

### 2.5. Histopathological Evaluation

All lesions resected were confirmed to be hemangioblastomas via histological microscopic inspection. Lesions with doubtful diagnosis were excluded. We also inspected the mitotic count and the KI-67 if available.

### 2.6. Patient Follow-Up

A minimum follow-up period of six months was a key inclusion criterion for our study. However, most patients were followed for minimum of 12 months. Patients underwent a neurological evaluation using the modified McCormick Scale (MMCS or ∆ MCS), defined as the difference between pre-operative and post-operative score. Consequently, MMCS < 1 corresponds to patients neurologically similar or improved after surgery, whereas MMCS ≥ 1 means a neurological deterioration postoperatively. Post-operative complications, if present, were reported. The presence of tumor as well as the use of adjuvant therapy were also accounted for in this study.

### 2.7. Statistical Analysis

Univariate analyses and associations between variables were calculated using the chi-square test for categorical and semi-categorical variables. Give the small sample size, Yates’s correction for continuity was applied to improve accuracy and avoid overestimating the relationship. Wilcoxon signed-rank test was used to calculate differences for continuous variables (given the small sample size, normal distribution of data was not assumed). All statistical tests were two-sided, and the local significance level was set at 0.05. Statistical tests were carried out to confirm previously generated hypotheses, so the resulting *p*-values are not to be considered exploratory (i.e., no adjustment for multiple testing needed). All statistical analyses were performed using the R4.1.1.

## 3. Results

The cohort’s characteristics at baseline are summarized in Table 2.

### 3.1. Patient Population

In this international multicentric study, a total of 35 patients who underwent surgical resection for histologically confirmed spinal hemangioblastomas were included according to the inclusion and exclusion criteria. The median age of 52 (Q1–Q3 range, 34.5–60) was recorded. Twenty-one (60%) of patients were males, and fourteen (40%) were females. The median follow-up period was 37.5 months (12–75), and the mean duration of symptoms before surgery was 25 ± 38.7 months.

### 3.2. Clinical Manifestations (Table 2)

The pre-operative clinical assessment showed a predominant Frankel score D represented in 22 patients (66.7%), followed by a Frankel score E found in 10 patients (30.3%). McCormick score of II was the most common at 60.6%, followed by a McCormick score of I at 27.3%. A moderate McCormick score III was reported in 12.1% of patients. No patients presented with a McCormick IV or V in the pre-operative evaluation. While most patients had a combination of signs of symptoms at the initial evaluation, sensory disturbance was found in 28 patients, making it the most frequent clinical feature at 82.4%. Around one-third of the of patients experienced pain in the pre-operative period at 35.3%. Motor deficits were found in 50% of patients. Gait ataxia was relatively less common and seen in the 35.3% of patients. Sphincter disturbance was the least frequent clinical manifestation at only 5.7%. Two patients were completely asymptomatic, presenting in 5.7% of cases.

### 3.3. Radiological Features (Table 2)

Lesions were most commonly located in the cervical region at 54.3%, whereas 31.4% of tumors were found the thoracic region, making it the second most common spinal level in our series. The cervicothoracic segment and the thoracolumbar segment were less common, found in 8.6% and 2.9%, respectively. Only one tumor was found the in lumbar region (2.9%). Tumor dimensions were expressed in cranio-caudal diameter (height), transversal diameter (width), and antero-posterior diameter (depth). Twenty-eight tumors were purely intramedullary with a percentage of 80%, while five (14.3%) were purely extramedullary. Two lesions had a combined intra-extra medullary form at 5.7%. Syringomyelia (cystic component) was identified in 72% of cases. With regards to their transversal locations, more than half were found to be dorsal or dorsolateral (55%). A ventral or ventro-lateral location was observed in 25% of cases, while 20% were central or centro-lateral.

### 3.4. Surgical Data, IONM Findings, and Complications (Table 3)

We found the mean surgical duration to be at 268.4 min ± 104.5. While a complete surgical resection (GTR) was achievable for the vast majority (88.2%), four patients underwent sub-total resection (11.8%).

**Table 3:** Operative data, IONM findings, complications, mortality, and recurrence rates.

	Total Population, n = 35
**I Surgical data**	
Operative duration (min), mean ± SD	268 ± 104
Type of resection, n (%)	
Gross total resection (GTR)	30 (88.2) ^a^
Sub-total resection (STR)	4 (11.8) ^a^
* Missing data*	*1*
**II Intra-operative neuromonitoring (IONM) changes, n (%)**	
SSEP	2 (8.7) ^b^
MEP	3 (13.0) ^b^
D-wave	5 (21.7) ^b^
* Missing data*	*12*
**III Type of complications, n (%)**	
Neurological deterioration (immediate)	11 (33.3) ^c^
Neurological deterioration (last follow-up)	3 (10.3) ^d^
CSF leak	3 (8.6)
Surgical site infection	1 (2.9)
Hematoma	1 (2.9)
Spinal instability	1 (2.9)
**IV Mortality, n (%)**	0 (0.0)
**V Recurrence, n (%)**	3 (8.6)

^a^ percentage among n = 34; ^b^ percentage among n = 23; ^c^ percentage among n = 33; ^d^ percentage among n = 29.

Intra-operative monitoring via motor and sensory evoked potentials was used for 23 patients. Among those, changes were observed in two patients for SEP and three for MEP (8.7 and 13% respectively). D-wave registration showed intra-operative changes for five patients (21.7%).

Including neurological deterioration at last follow-up, post-operative complications were reported in nine (25.7%) patients. The presence of a CSF leak was one of the most common complications at 30%. Post-operative infections, hemorrhages, and spinal stability were less frequent at 11.1% for each. No surgery-related deaths during hospital stay were reported in the series.

### 3.5. Histopathology

Pathological diagnosis of hemangioblastoma was confirmed for all specimens. The presence of mitosis was inspected in more than half of cases and was found to be <1 in all. The KI-67 index was inspected in just over one-third of cases. This was found to be at 3% in 20% of cases inspected, 1–2% in 38.5%, and <1% in 41.5% of cases.

### 3.6. Clinical Outcome (Table 4)

A good overall McCormick score (I–II) was observed in the last follow-up in 82% of patients. The majority of patients showed a good MCS (I–II) in the immediate post-operative evaluation (66.7%). A moderate to severe MCS (III–V) were reported in 33.3% in the immediate post-operative evaluation compared to 22% one month after surgical intervention. However, this was further reduced in the last follow-up visit (18%). Just over a half of patients (51.5%) were discharged home, and the rest (48.5%) were sent to rehabilitation centers.

**Table 4:** Clinical outcome after surgery (descriptive results).

	Total Population, n = 35
**I Type of discharge, n (%)**	
Home	17 (51.5) ^a^
Rehabilitation center	16 (48.5) ^a^
* Missing data*	*2*
**II McCormick Scale, n (%)**	
Immediate post-operative period	
Grade I	7 (21.2) ^a^
Grade II	15 (45.5) ^a^
Grade III	5 (15.2) ^a^
Grade IV	5 (15.2) ^a^
Grade V	1 (3.0) ^a^
* Missing data*	*2*
At 1 month postoperatively	
Grade I	7 (25.9) ^b^
Grade II	14 (51.9) ^b^
Grade III	3 (11.1) ^b^
Grade IV	2 (7.4) ^b^
Grade V	1 (3.7) ^b^
* Missing data*	*8*
At 6 months postoperatively	
Grade I	10 (40.0) ^c^
Grade II	11 (44.0) ^c^
Grade III	1 (4.0) ^c^
Grade IV	2 (8.0) ^c^
Grade V	1 (4.0) ^c^
* Missing data*	*10*
At last follow-up	
Grade I	11 (39.3) ^d^
Grade II	12 (42.9) ^d^
Grade III	3 (10.7) ^d^
Grade IV	1 (3.6) ^d^
Grade V	1 (3.6) ^d^
* Missing data*	*7*

^a^ percentage among n = 33; ^b^ percentage among n = 27; ^c^ percentage among n = 25; ^d^ percentage among n = 28.

### 3.7. Relative Outcome and Comparative Analysis (Table 5)

In order to identify clinical variables associated with a good overall post-operative outcome, a univariate statistical analysis was conducted between patients who showed a positive (unchanged or improved) ∆ MCS and patients who showed a negative (worsening) ∆ MCS in the immediate post-operative evaluation as well as in the 1-month, 6-month, and last follow-up evaluations.

**Table 5:** Statistical analysis regarding neurological outcome at immediate post-operative period (**I**), 1 month (**II**), 6 months (**III**), and last follow-up (**IV**) versus pre-operative status.

**I- Immediate Post-Operative Period (n = 32)**			
	**Modified McCormick ≤ 0** **(Unchanged or Improved)**	**Modified McCormick > 0** **(Deteriorated)**	***p*-value**
Number of patients (%)	21 (65.6)	11 (34.4)	-
Age, mean [Q1–Q3]	41 [29–52]	56 [51–61]	**0.03**
Sex, n (%)			
F	10 (47.6) ^a^	4 (36.4) ^b^	ns
M	11 (52.4) ^a^	7 (63.6) ^b^	
Tumor dimensions (mm), mean ± SD			
Cranio-caudal	17 ± 14	13 ± 9	ns
Transversal	11 ± 6	9 ± 3	ns
Antero-posterior	11 ± 11	11 ± 5	ns
Symptoms duration (months), mean ± SD	14.8 ± 23	28.1 ± 31	ns
Intra/extramedullary location, n (%)			
IM	16 (76.2) ^a^	10 (90.9) ^b^	ns
EM	5 (23.8) ^a^	1 (9.1) ^b^	
Associated syrinx, n (%)			
No	6 (30) ^c^	3 (30) ^d^	ns
Yes	14 (70) ^c^	7 (70) ^d^	
Frankel grade, n (%)			
C	0 (0.0) ^a^	1 (9.1) ^b^	ns
D	14 (66.7) ^a^	7 (63.6) ^b^	
E	7 (33.3) ^a^	3 (27.3) ^b^	
Type of resection, n (%)			
GTR	18 (85.7) ^a^	10 (90.9) ^b^	ns
STR	3 (14.3) ^a^	1 (9.1) ^b^	
IONM changes, n (%)			
SSEP changes			
No	13 (86.7) ^e^	7 (100) ^f^	ns
Yes	2 (13.3) ^e^	0 (0.0) ^f^	
MEP changes			
No	14 (93.3) ^e^	5 (71.4) ^f^	ns
Yes	1 (6.7) ^e^	2 (28.6) ^f^	
D-wave changes			
No	10 (100) ^g^	0 (0.0) ^h^	**0.015**
Yes	0 (0.0) ^g^	2 (100) ^h^	
Complications, n (%)			
No	19 (90.5) ^a^	9 (81.8) ^b^	ns
Yes	2 (9.5) ^a^	2 (18.2) ^b^	
Duration of surgery (min), mean ± SD	245 ± 67	300 ± 132	ns
Length of hospital stay (days), mean ± SD	9.9 ± 4.8	15.2 ± 10	ns
Type of discharge, n (%)			
Home	15 (71.4) ^a^	2 (18.2) ^b^	**0.013**
Rehabilitation center	6 (28.6) ^a^	9 (81.8) ^b^	
^a^ percentage among n = 21; ^b^ percentage among n = 11; ^c^ percentage among n = 20; ^d^ percentage among n = 10; ^e^ percentage among n = 15; ^f^ percentage among n = 7; ^g^ percentage among n = 10; ^h^ percentage among n = 2			
**II- At 1 month postoperatively (n = 27)**			
	**Modified McCormick ≤ 0** **(Unchanged or improved)**	**Modified McCormick > 0** **(Deteriorated)**	***p*-value**
Number of patients (%)	19 (70.4)	8 (29.6)	-
Age, mean [Q1–Q3]	39 [29–50]	59 [56–65]	**0.01**
Sex, n (%)			
F	9 (47.4) ^a^	4 (50.0) ^b^	ns
M	10 (52.6) ^a^	4 (50.0) ^b^	
Tumor dimensions (mm), mean ± SD			ns
Cranio-caudal	18 ± 15	12 ± 10	ns
Transversal	10 ± 6	10 ± 2	ns
Antero-posterior	11 ± 11	11 ± 4	ns
Symptoms duration (months), mean ± SD	19.9 ± 30	43.2 ± 64	ns
Intra/extramedullary location, n (%)			
IM	14 (73.7) ^a^	7 (87.5) ^b^	ns
EM	5 (26.3) ^a^	1 (12.5) ^b^	
Associated syrinx, n (%)			
No	5 (27.8) ^c^	3 (37.5) ^b^	ns
Yes	13 (72.2) ^c^	5 (62.5) ^b^	
Frankel grade, n (%)			
C	0 (0.0) ^a^	0 (0.0) ^b^	ns
D	14 (73.7) ^a^	5 (62.5) ^b^	
E	5 (26.3) ^a^	3 (37.5) ^b^	
Type of resection, n (%)			
GTR	16 (84.2) ^a^	7 (87.5) ^b^	ns
STR	3 (15.8) ^a^	1 (12.5) ^b^	
IONM changes, n (%)			
SSEP changes			
No	11 (91.7) ^d^	7 (100) ^e^	ns
Yes	1 (8.3) ^d^	0 (0.0) ^e^	
MEP changes			
No	11 (91.7) ^d^	5 (71.4) ^e^	ns
Yes	1 (8.3) ^d^	2 (28.6) ^e^	
D-wave changes			
No	8 (100) ^b^	0 (0.0) ^f^	**0.01**
Yes	0 (0.0) ^b^	3 (100) ^f^	
Complications, n (%)			
No	16 (84.2) ^a^	7 (87.5) ^b^	ns
Yes	3 (15.8) ^a^	1 (12.5) ^b^	
Duration of surgery (min), mean ± SD	249 ± 59	322 ± 148	ns
Length of hospital stay (days), mean ± SD	10.3 ± 4.8	15.4 ± 11.1	ns
Type of discharge, n (%)			
Home	12 (63.2) ^a^	2 (25.0) ^b^	ns
Rehabilitation center	7 (36.8) ^a^	6 (75.0) ^b^	
^a^ percentage among n = 19; ^b^ percentage among n = 8; ^c^ percentage among n = 18; ^d^ percentage among n = 12; ^e^ percentage among n = 7; ^f^ percentage among n = 3			
**III- At 6 months postoperatively (n = 25)**			
	**Modified McCormick ≤ 0** **(Unchanged or improved)**	**Modified McCormick > 0** **(Deteriorated)**	***p*-value**
Number of patients (%)	19 (76.0)	6 (24.0)	-
Age, mean [Q1–Q3]	41 [32–54]	56.5 [55–59]	ns
Sex, n (%)			
F	9 (47.4) ^a^	2 (33.3) ^b^	ns
M	10 (52.6) ^a^	4 (66.7) ^b^	
Tumor dimensions (mm), mean ± SD			
Cranio-caudal	17 ± 15	15 ± 10	ns
Transversal	11 ± 6	11 ± 2	ns
Antero-posterior	11 ± 12	14 ± 1	ns
Symptoms duration (months), mean ± SD	19.3 ± 30	58 ± 71	ns
Intra/extramedullary location, n (%)			
IM	14 (73.7) ^a^	5 (83.3) ^b^	ns
EM	5 (26.3) ^a^	1 (16.7) ^b^	
Associated syrinx, n (%)			
No	5 (27.8) ^c^	2 (33.3) ^b^	ns
Yes	13 (72.2) ^c^	4 (66.6) ^b^	
Frankel grade, n (%)			
C	0 (0.0) ^a^	0 (0.0) ^b^	ns
D	14 (73.7) ^a^	3 (50.0) ^b^	
E	5 (26.3) ^a^	3 (50.0) ^b^	
Type of resection, n (%)			
GTR	16 (84.2) ^a^	5 (83.3) ^b^	ns
STR	3 (15.8) ^a^	1 (16.7) ^b^	
IONM changes, n (%)			
SSEP changes			
No	11 (91.7) ^d^	5 (100) ^e^	ns
Yes	1 (8.3) ^d^	0 (0.0) ^e^	
MEP changes			
No	11 (91.7) ^d^	4 (80.0) ^e^	ns
Yes	1 (8.3) ^d^	1 (20.0) ^e^	
D-wave changes			
No	8 (100) ^f^	0 (0.0) ^g^	**0.03**
Yes	0 (0.0) ^f^	2 (100) ^g^	
Complications, n (%)			
No	16 (84.2) ^a^	5 (83.3) ^b^	ns
Yes	3 (15.8) ^a^	1 (16.7) ^b^	
Duration of surgery (min), mean ± SD	264.6 ± 69	334 ± 148	ns
Length of hospital stay (days), mean ± SD	10 ± 5	17 ± 12	**0.04**
Type of discharge, n (%)			
Home	11 (57.9) ^a^	2 (33.3) ^b^	ns
Rehabilitation center	8 (42.1) ^a^	4 (66.6) ^b^	
^a^ percentage among n = 19; ^b^ percentage among n = 6; ^c^ percentage among n = 18; ^d^ percentage among n = 12; ^e^ percentage among n = 5; ^f^ percentage among n = 8; ^g^ percentage among n = 2			
**IV- At last follow-up (n = 25)**			
	**Modified McCormick ≤ 0** **(Unchanged or improved)**	**Modified McCormick > 0** **(Deteriorated)**	***p*-value**
Number of patients (%)	22 (88.0)	3 (12.0)	-
Age, mean [Q1–Q3]	41.5 [30–55]	56 [41–59]	ns
Sex, n (%)			
F	11 (50.0) ^a^	0 (0.0) ^b^	ns
M	11 (50.0) ^a^	3 (100) ^b^	
Tumor dimensions (mm), mean ± SD			
Cranio-caudal	17 ± 14	11 ± 7	ns
Transversal	10 ± 6	12 ± 1	ns
Antero-posterior	11 ± 11	15 ± 0	ns
Symptoms duration (months), mean ± SD	20 ± 29	24 ± 30	ns
Intra/extramedullary location, n (%)			
IM	16 (72.7) ^a^	3 (100) ^b^	ns
EM	6 (27.3) ^a^	0 (0.0) ^b^	
Associated syrinx, n (%)			
No	7 (33.3) ^c^	1 (33.3) ^b^	ns
Yes	14 (66.7) ^c^	2 (66.7) ^b^	
Frankel grade, n (%)			
C	0 (0.0) ^a^	1 (33.3) ^b^	**0.02**
D	16 (72.7) ^a^	1 (33.3) ^b^	
E	6 (27.3) ^a^	1 (33.3) ^b^	
Type of resection, n (%)			
GTR	19 (86.4) ^a^	2 (66.7) ^b^	ns
STR	3 (13.6) ^a^	1 (33.3) ^b^	
IONM changes, n (%)			
SSEP changes			
No	13 (92.9) ^d^	3 (100) ^b^	ns
Yes	1 (7.1) ^d^	0 (0.0) ^b^	
MEP changes			
No	12 (85.7) ^d^	3 (100) ^a^	ns
Yes	2 (14.3) ^d^	0 (0.0) ^a^	
D-wave changes			
No	8 (88.9) ^e^	NA	ns
Yes	1 (11.1) ^e^	NA	
Complications, n (%)			
No	18 (81.8) ^a^	3 (100) ^b^	ns
Yes	4 (18.2) ^a^	0 (0.0) ^b^	
Duration of surgery (min), mean ± SD	270 ± 85	253 ± 50	ns
Length of hospital stay (days), mean ± SD	11 ± 5	26 ± 23	**0.02**
Type of discharge, n (%)			
Home	13 (59.1) ^a^	1 (33.3) ^b^	ns
Rehabilitation center	9 (40.9) ^a^	2 (66.7) ^b^	
^a^ percentage among n = 22; ^b^ percentage among n = 3; ^c^ percentage among n = 21; ^d^ percentage among n = 14; ^e^ percentage among n = 9			

Statistical significance was considered for *p*-value < 0.05.

Advanced age was found to be associated with a worsening clinical outcome in the immediate post-operative and the one-month evaluations (*p* = 0.03 and 0.01, respectively). However, this was not found to be of statistical significance in the 6-month and the last follow-up evaluations.

Sex and previous surgeries did not have any significant impact of the post-operative outcome.

Neither the affected spinal level nor the number of segments involved showed and had any significant statistical effect on the overall outcome. Moreover, tumor size and topographic location as well as the presence of syringomyelia were not of statistical significance.

Although pre-operative DSA was used for around one-third of patients, this did not have any significant statistical effect in post-operative evaluation.

Even though changes in intra-operative MEP or SEP were not associated with any statistical significance, absence of D-wave changes intra-operatively was associated a positive outcome (*p* < 0.05 at immediate post-operative, 1-month, and 6-month evaluations).

Hospitalization duration was not found to be of statistical significance in the short term. However, longer hospital stays were associated a worse long-term outcome, as demonstrated in the 6-month and last follow-up evaluations (*p* = 0.042 and 0.15, respectively).

Clinical neurological outcome was not affected by the duration of the surgical procedure or tumor recurrence.

Overall, these data suggest that most patients showed long-term post-operative improvement. They also suggest that the overall outcome may be affected by patients’ ages and D-wave changes during intra-operative monitoring.

## 4. Discussion

This study offers a relatively large case series (n > 30) for lesions that are considered rare. Moreover, by providing an international multicentric study, a non-biased variable clinical experience is illustrated regarding to the short- and long-term post-operative outcomes for these rare lesions.

Spinal hemangioblastomas are more common among middle-aged adults according to the literature, with the suggested median patient age found in the literature was 44.5 years, as reported by Jankovic et al. in their recent systemic review [2]. Our results were consistent with this fact. The median age patient age recorded herein was at 52 years. In our experience, we found that patients’ age was a reliable factor for predicting the neurological outcome for the immediate post-operative period and short-term follow-up. However, this was proven unreliable for follow-up extending beyond six months. A large series of 92 patients published by Deng et al. showed similar results. They illustrated that age should not be considered as a reliable factor for predicting post-operative outcomes [12].

These tumors may exhibit slight male predominance. According to the literature, the recorded male-to-female ratios vary from 1.6:1 to 5.5:1 [2,12,14,25]. We also found a slight male predominance at 58.8%. Similar results showing a slight male predominance at 53% were reported by Sadashivam et al. in their series published in 2020 [26]. However, this was not consistent in the literature, and some exceptions were found. For instance, Mandigo et al. reported eight females and seven males in their series published in 2009, and Jang et al. reported a 68% female predominance [1,14]. Despite this slight male predominance, it did not have an impact on the overall clinical outcome and is therefore not a reliable predictable factor.

Clinical presentations depend on the size and location of lesions as well the presence of syringomyelia and spinal cord edema [2,5,8,14]. The most common clinical presentation reported in recent reviews is sensory deficit [1,3,12]. Jankovic et al. reported pain as the most common symptom at 34.5% and sensory deficit as the most common neurological sign at 28.1%. Similarly, Deng et al. found sensory deficits and pain to be the most frequently encountered clinical manifestations in their large series of 92 cases at 65.2% and 53.2%, respectively [12]. These data are consistent with our findings seeing that we observed that sensory deficits were the most common form of presentation. This was followed by motor deficit, gait ataxia and pain respectively. Sphincter disturbances were the least common clinical feature in this study.

It was also noticed that most patients had a good long-term post-operative evolution. An improvement in the ∆ MCS is noted in the 1 month post-operative period compared to the immediate post-operative evaluation for a majority of patients (>70%). This has further improved in the 6-month evaluation and reached nearly 90% of the cohort at the last follow-up. Patients that showed a lack of significant improvement in the long term already exhibited a severe MMCS in the immediate post-operative assessment. Most patients with a MMCS of IV or V postoperatively did not return to their baseline examination.

Regarding HBs location, the cervical region is frequently reported the most common tumoral localization in the literature, followed by the thoracic then the lumbar region [6,12,16]. For instance, 48% of cases reported by Mossel et al. were located in the cervical region [6]. Our results were consistent with these facts. We found that more than half of tumors were in the cervical region, and just under one-third were in the thoracic segment. About the topography into the transversal plane, a dorsal tumoral location within the spinal cord was reported as the most frequent and with the most favorable outcome. This is probable due to the facility of surgical access [1,3,27,28,29]. We also noticed the dorsal location to be the most common. Indeed, more than half of the images inspected in this series showed a dorsal or a dorso-lateral location in relation to the spinal cord. However, we did not notice any statistical significance in the clinical outcome compared to other location in relation to the spinal cord. Variation in tumor sizes have also been inspected in the literature as a prognostic factor, and results vary. For instance, Jang et al. found tumoral sizes to be a statistically significant prediction factor [1]. On the other hand, similar to Deng et al., we found tumor dimension to be unreliable for predicting outcome [12]. Herein, there was no impact of tumor dimensions on overall clinical outcome.

Cystic formations are quite frequent in spinal tumors, and HBs are no exception. HBs are frequently associated with cystic formations in the form of syringomyelia, which is present in more than 50% of cases according to the literature [8,30]. Jang et al. reported the presence of syringomyelia in 96% of cases in their recent publication. However, they did not find that the presence of syrinx significantly influences the post-operative outcome [1]. Despite being found in more than two-thirds of patients we reported, we did not find them to have a significant effect on the overall outcome.

The use of intra-operative neuro-monitoring is becoming frequent nowadays when dealing with spinal tumors. It is considered a tool to prevent operative complications as well as predict the neurological outcome [8]. Several authors have found those results to be reliable predicting factors, such as Feletti et al. [8,31]. We found MEP and SEP changes to be of no significance in predicting outcome. However, D-wave changes were found to be a significant reliable factor. While patients having experienced MEP and SEP changes generally recovered well, those having D-wave changes experienced long-lasting neurological manifestations contributing to a negative ∆ MCS.

Although advancement in the treatment of HBs, such stereotaxic radiosurgery, have been introduced, surgical resection remains the gold standard of treatment [20]. Concerning the surgical technique, patients are positioned in a prone position and maintained in neutral position via Mayfield head clamps, and the desired level is confirmed with X-ray images prior to skin incision. The surgical treatment typically consists of midline surgical incision and dissection of paraspinous muscles to the level of laminae and spinous processes, followed by a laminectomy, a laminotomy, or a laminoplasty based on the surgeon’s preference. The dura is opened next via a vertical incision and retained laterally with sutures. A dorsal midline myelotomy is then carried out, exposing the tumor. Feeding arteries and draining veins should identified and coagulated, and the tumor should be removed en bloc when possible. Penetrating the lesion must be absolutely avoided, seeing that HBs are vascular lesions with high risk of massive intra-operative bleeding. Modern techniques such fluorescence can be used for better visualization of the tumor and for more precise identification of feeding and draining vessels. Surgery should be carried out under IONM when available and, ideally, with D-wave monitoring [8,12,14,27]. Complete surgical resection is usually achievable, with partial resections being exceptional. As illustrated by Jankovic et al. in their relatively recent systematic review, gross total resection was achieved in 83.52% of cases, while subtotal resections and partial resections were seen in only 9.17% and 3.5%, respectively. They also reported a good surgical tolerance with a favorable overall outcome seen in 77% of cases [2]. In our experience, we found that a gross total resection was achievable in nearly 90% of cases, with a relatively good overall outcome. However, the extent of resection of did not have a statistically significant effect of the post-operative development, even if the risk of local recurrence is considered. Complication rates are seemingly low internationally. Jankovic et al. and Deng et al. reported a 6.2% and 8.7% complication rate, respectively. Each reported CSF leaks to be the most common complication. In the present series, we found complications to be significantly more frequent, found in up to 25.7% of cases, and CSF leaks were the most common at 30%. Nonetheless, these did not have a significant impact on the clinical outcome from a statistical standpoint.

Von Hipple–Lindau is a genetic condition with devastating neoplastic manifestations affecting multiple organs, such renal cell carcinoma and pancreatic carcinoma. CNS neoplasia is also seen in association with this condition, such as intracranial and spinal hemangioblastomas and retinoblastoma [19,23,32]. This condition is inherited in an autosomal dominant fashion and characterized by a germline mutation in the VHL gene located in the chromosome 3p25–26 region [6,10]. Spinal HBs account for around 40% of CNS lesions associated with VHL, making them the second most common after intracranial hemangioblastomas. On the other hand, 20–30% of all spinal hemangioblastomas are associated with VHL [3,8,11,12,13,23]. Despite surgery being the gold standard for treatment, non-surgical alternatives have been emerging in recent years. Belzutifan (MK-6482) is a new second-generation small-molecule inhibitor of hypoxia-inducible factor 2α (HIF-2α) and is proposed as a specific treatment for VHL-induced tumors such hemangioblastoma. Positive results have been seen in clinical trials so far, and an FDA approval was obtained recently [21,32].

Another alternative treatment to surgical intervention that has been discussed in recent years is the use of stereotaxic radiosurgery. In their retrospective analysis of nine patients treated for spinal hemangioblastomas via stereotaxic radiosurgery, Selch et al. found that the overall local control and solid tumor control rates at 48 months were 90% and 95%, respectively, and only one patient needed surgical resection for worsening pain [33]. Similar results were reported by Cvek et al. in their study, where a 27–33% volume reduction was observed following radiosurgery, with no reported complications [20]. This shows that stereotaxic radiosurgery could considered as an alternative to surgery.

To the best of our knowledge, only 10 series with more than 30 patients have been published regarding the surgical treatment of spinal hemangioblastomas. The prognostic factors found from these series are presented in Table 6. Lonser et al. showed the clinical outcome was most affected by the tumor’s antero-posterior location in relation to the dentate ligament. A worse outcome was linked to an anterior location, probably related to the need for spinal cord manipulation in such a situation. They also illustrated that a large tumor volume could be associated with a worse outcome [27]. Similarly, Kanno et al. found larger tumors to associated with a worsening outcome as well [23]. In our study, we did not observe correlation between tumor size and clinical outcome. Von Hipple–Lindau was of interest in the paper published by Parker et al. They showed that the VHL was not associated with worsening immediate or short post-operative evolution, but it was associated with a worsening long-term outcome due to the appearance of other lesions and/or multiple surgeries [34]. Similar to Lonser et al., Mehta et al. found that tumors situated anterior to the dentate ligament were associated with decline in post-operative outcome in addition to lesions that were completely intramedullary and the use of myelotomy to facilitate surgical resection [13]. Furthermore, Takai et al. found the patient’s age at surgery could affect the surgical outcome, similar to our results. They also found a correlation between the number of lesions resection in one surgical session and post-operative decline as well as partial surgical resections [35]. Westwick et al. reported advanced age over 75 years to affect the overall survival. However, they claimed this to be related to death because of morbidity related to old age rather than post-operative evolution [4]. Feletti et al. showed a special interest in the use of intra-operative neuromonitoring. Their results showed a favorable outcome related to the use IONM compared to other cases. They also showed that the use of a laminectomy rather than a laminotomy was related to poorer outcome [8]. Intramedullary lesions were also related to a poorer outcome according to Butenschoen et al., as was also found by Mehta et al. Butenschoen also found that a poor pre-operative MMSC was associated with a poor outcome and vice versa. Interestingly, they also found laminoplasties to be predictors of a poorer outcome compared to laminectomies; however, this was only shown in univariate analysis and was not exhibited in multivariate analysis. In addition, they declared partial resections as a poor prognostic factor in long term follow-up, probably due to tumor recurrence [15].

### 4.1. Illustrative Case

A 40 year-old male, known as a schizophrenic person, presented with neck pain and right-sided sensory disturbance for 1 year. He also complained of mild instability when walking and a decrease in power in the side of body.

Physical examination revealed a motor deficit with a score of 4/5 on the right upper and lower limbs, associated a right-sided sensory deficit without any upper motor neuron signs. Pre-operative Frankel score D and McCormick score II were assigned.

**Figure 1:**
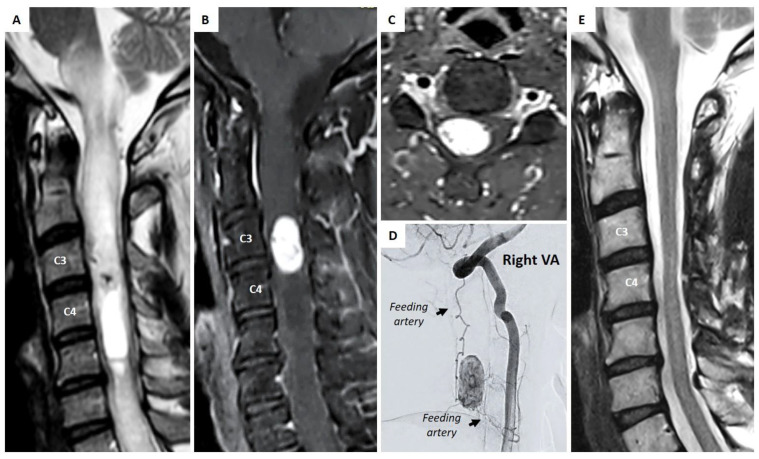
Hemangioblastoma in the cervical spine at C3–C4. A caudal satellite cyst was clearly visible with extensive intra-medullary oedema up to the medulla oblongata on sagittal T2-weighted MR sequence (**A**). The solid portion of the lesion was well delimited and characterized by an hyperintense enhancement on T1-weighted gadolinium MR sequence (**B**,**C**). Angiography demonstrated the hypervascularity of the lesion with tumoral blush and visualization of several feeding arteries (**D**). Post-operative MRI at 10 months confirmed the radical resection of the lesion with much better nice aspect of the spinal cord, no residual tumor, and absence of post-operative complication (**E**).

Pre-operative MRI showed a intramedullary C3–C4 enhancing lesion with associated syringomyelia and surrounding spinal cord edema extending to the cranio-cervical junction cranially and C7 caudally, with high gadolinium enhancement (Figure 1A–C). DSA was also realized and showed feeding arteries originating from the V2, V3, and V4 segments from the right vertebral artery as well as the ascending cervical artery on the right side. Venous drainage was via the anterior and posterior spinal veins (Figure 1D).

The pre-operative evoked potential revealed abnormal results on both MEP and SEP, but the sensory anomaly was more prominent.

**Figure 2:**
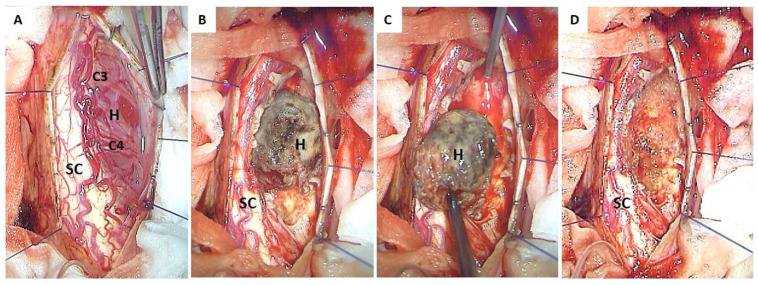
Intra-operative views. The tumor looked highly vascularized with abundant vascular structures on its surface (**A**). Coagulation of feeding vessels at the cord–tumor interface was first achieved. The resection was then conducted progressively without penetrating inside the tumor and occurred at the end of radical complete en bloc removal (**B**,**C**). The spinal cord was carefully respected during all the surgical procedure and meticulously preserved with the assistance of IONM (**D**). *H*—*hemangioblastoma*. *SC*—*spinal cord*.

Surgical intervention was carried out. Access was granted via a laminoplasty on C3–C4, with a partial laminectomy on C2 and C5. The dura was opened next via vertical incision and retained laterally using 5.0 sutures. This was followed by opening of the arachnoid underneath and fixing it to the dural edges using hemoclips. The tumor was clearly visualized at this point, with its right posterolateral position in relation to the spinal cord (Figure 2A). Feeding vessels were identified and coagulated before starting tumor dissection from the surrounding spinal cord (Figure 2B). En bloc tumor resection was achievable at the end of the surgery (Figure 2C,D). The dura was closed afterwards in watertight fashion, followed by progressive closure of the overlying outmost structures. No changes were detected during the intra-operative monitoring.

The immediate post-operative evaluation found worsening of the neurological deficit, with a motor power of 1/5 in the right upper and lower limbs. This progressively improved in a few days during the hospital stay to 3/5 in the right upper limb and 4/5 in right lower limbs, with a McCormick score of II. The 1-month post-operative evaluation revealed a motor deficit of 4/5 in the right upper and lower limbs, returning to baseline with a modified McCormick score II.

The 6-month and last follow-up evaluation both showed an almost complete resolution of the motor deficit, estimated to 4+/5, with the persistence of fine motor difficulty in the right hand and sensory deficit in the right upper limb. A complete resolution was noted in the right lower limb, corresponding finally to a McCormick score I.

The immediate post-operative MRI confirmed the complete resection and the follow-up MRI performed 3 months postoperatively showed no signs of residual tumor (Figure 1E).

### 4.2. Limitation

Despite the multicentric nature of this study and the relatively large number of cases compared to the vast majority of published series, the main limitation of the present study is represented by its retrospective design. Also, we could not properly evaluate the effect of VHL on the post-operative outcome since most patients were not genetically tested, and this could be considered another weakness of this study being multicentric. Finally, having treated all the patients surgically, we could not test the efficacy of alternative treatment methods such as radiosurgery.

## 5. Conclusions

Even if spinal HBs are benign intra/extra-medullary lesions, they may result in significant neurological deficits affecting patients’ quality of the life. Surgical resection for symptomatic lesions seems to be an appropriate treatment and is associated with a good overall post-operative outcome after surgery, with unchanged or improved neurological status in the majority of patients. The significant factors associated with a poorer neurological outcome were the advanced age and D-wave changes during IONM. Other factors, such as tumor dimensions, location, and/or the presence of syrinxes, did not significantly impact the neurological outcome. Finally, the aim of the surgical treatment should be gross total resection, which is achievable for most patients. The surgery of these vascular lesions with no possibility of debulking or piece-meal removal and requiring “en bloc” resection is technically demanding and should only be performed by teams experienced in spine and spinal cord surgery.

## Figures and Tables

**Table 1:** Frankel (1) and modified McCormick (2) scales utilized in the present study for pre- and post-operative neurological assessment.

Grade	Clinical Significance
**1- Frankel**	
A	Complete neurological injury—No motor or sensory function detected below level of lesion
B	No motor function detected below level of lesion—some sensory function preserved
C	Some voluntary motor function preserved but too weak to serve any useful purpose
D	Functionally useful voluntary motor function below level of injury is preserved
E	Normal motor and sensory function below level of lesion—abnormal reflexes may persist
**2- McCormick**	
I	Neurologically intact, ambulates normally, may have minimal dysesthesia
II	Mild motor or sensory deficit; patient maintains functional independence
III	Moderate deficit, limitation of function, independent with external aid
IV	Severe motor or sensory deficit, limit of function with a dependent patient
V	Paraplegic or quadriplegic, even if there is flickering movement

**Table 2:** Demographical, clinical and radiological cohort’s characteristics at baseline (n = 35).

	Total Population, n = 35
**I Demographic data**	
Total of patients, n (%)	35 (100)
Age (years), mean [Q1–Q3]	52 [34.5–60]
Male, n (%)	21 (60)
**II Clinical presentation**	
Symptoms duration (months), mean ± SD	25 ± 38.7
Type of symptoms, n (%)	
Sensory disturbance	28 (82.4) ^a^
Motor deficit	17 (50.0) ^a^
Ataxia	12 (35.3) ^a^
Pain	12 (35.3) ^a^
Bladder disfunction	2 (5.9) ^a^
* Missing data*	*1*
Frankel score at baseline, n (%)	
A	0 (0) ^b^
B	0 (0) ^b^
C	1 (3.0) ^b^
D	22 (66.7) ^b^
E	10 (30.3) ^b^
* Missing data*	*2*
Modified McCormick score at baseline, n (%)	
Grade I	9 (27.3) ^b^
Grade II	20 (60.6) ^b^
Grade III	4 (12.1) ^b^
Grade IV	0 (0) ^b^
Grade V	0 (0) ^b^
* Missing data*	*2*
**III Radiological features**	
Location, n (%)	
Cervical	19 (54.3)
Cervico-dorsal	3 (8.6)
Dorsal	11 (31.4)
Dorso-lumbar	1 (2.9)
Lumbar	1 (2.9)
Tumor dimensions (mm), mean ± SD	
Cranio-caudal	16 ± 12
Transversal	10 ± 5
Antero-posterior	11 ± 10
Number of involved levels, n (%)	
1–2 levels	24 (82.8) ^c^
≥3 levels	5 (17.2) ^c^
* Missing data*	*6*
Associated syrinx, n (%)	
Yes	23 (71.9) ^d^
No	9 (28.1) ^d^
* Missing data*	*3*
Transversal location, n (%)	
Ventral or ventro-lateral	5 (25.0) ^e^
Dorsal or dorso-lateral	11 (55.0) ^e^
Central	4 (20.0) ^e^
* Missing data*	*15*
Intra/extramedullary location, n (%)	
Intramedullary	28 (80.0)
Extramedullary	5 (14.3)
Intra-extramedullary	2 (5.7)

^a^ percentage among n = 34; ^b^ percentage among n = 33; ^c^ percentage among n = 29; ^d^ percentage among n = 32; ^e^ percentage among n = 20.

**Table 6:** Prognostic factors reported from the literature (case series ≥ 30 patients).

Author, Year	n	Sex	Age	Significant Prognostic Factors
Lonser et al., 2003 [27]	44	26M	34	1- Anterior location to dentate ligament was associated with a worse prognosis 2- Tumor volume < 500 mm^3^ associated with a better outcome
Kanno et al., 2009 [23]	45	21M	33.5	Tumor volume < 500 mm^3^ associated with a better outcome
Wang et al., 2008	68	-	36.6	-
Parker et al., 2009 [34]	34	15M	41	VHL did not affect the immediate post-operative or short-term outcome, but long-term outcome was worsened due disease complications, such as other neoplasms
Mehta et al., 2010 [13]	108	57M	33.8	1- Anterior location to dentate ligament associated with a worse prognosis 2- IM tumors associated with a worse prognosis compared to EM tumors 3- Myelotomy was associated with a worse outcome
Takai et al., 2010 [35]	35	20M	39	1- Number of lesions removed at one time affected the outcome 2- Advanced age at the time of resection associated with a worse outcome 3- Partial resection associated with a worse outcome
Deng et al., 2014 [12]	92	59M	32.75	-
Westwick et al., 2016 [4]	133	62M	48	Age > 75 years affected overall survival
Yousef et al., 2019	42	31M	44	-
Feletti et al., 2022 [8]	61	30M	35	1- Laminectomies associated with a poorer outcome compared to laminotomies 2- Use of IONM associated with a more favorable outcome
Butenschoen et al., 2023 [15]	60	33M	51	1- IM tumors associated with a worse prognosis compared to EM tumors 2- Poor pre-operative state associated with a poorer outcome 3- Laminoplasties associated with a poorer outcome compared to laminectomies 4- Partial resections associated with a poorer outcome
Present study	35	21M	52	1- Advanced age associated with a less favorable prognosis 2- D-wave changes during IONM associated with a poorer outcome

## Data Availability

The original contributions presented in this study are included in the article. Further inquiries can be directed to the corresponding author.
